# Does dose matter? Parental attendance in a preventive intervention for anxious and sad children

**DOI:** 10.1186/s40359-024-02234-2

**Published:** 2024-12-18

**Authors:** Elisabeth Valmyr Bania, Kristin Ytreland, Anne Mari Sund, Stian Lydersen, Simon Peter Neumer, Frode Adolfsen, Kristin Martinsen, Lene-Mari Potulski Rasmussen, Jo Magne Ingul

**Affiliations:** 1 The Regional Centre for Child and Youth Mental Health and Child Welfare - Central Norway, RKBU Midt-Norge, NTNU, Postbox 8905 MTFS, Trondheim, NO-7491 Norway; 2 The Center for Child and Adolescent Mental Health – Eastern and Southern Norway, Postboks 4623, 0405 Nydalen, Oslo, Norway; 3 The Regional Centre for Child and Youth Mental Health and Child Welfare – Northern Norway, RKBU Nord UiT Norges Arktiske Universitet, Tromsø, 9037 Norway; 4 Department of Psychology, University of Oslo, Forskningsveien 3A, Oslo, 0373 Norway

**Keywords:** Parental involvement, Emotional difficulties, Dose effect, Indicated, Preventive, School, Group, CBT

## Abstract

**Background:**

International studies show increasing prevalence of anxiety and depression among children. Parents are vital for children in all aspects of life, also in supporting their offspring in promoting better mental health, life skills and reducing emotional difficulties. Therefore, involving parents in interventions aimed at preventing development of anxiety and depression is natural. In treatment studies, targeted parental involvement has been difficult to prove effective. However, few existing studies investigate the effect of parental involvement in preventive interventions.

**Objective:**

We aimed to explore whether attendance influenced the change in child’s emotional symptoms post intervention and one-year later reported by parents.

**Method:**

Parents of children attending an indicated preventive intervention named EMOTION, who took part in a high parental involvement condition were included in this study (*n* = 385). High involvement entailed 5 parent group sessions. Using linear mixed models, we investigated whether attendance in the parent groups influenced the parent-reported levels of children’s emotional symptoms post-intervention and at one-year follow-up.

**Results:**

Parents who did not attend parent sessions reported significantly larger reductions in child anxiety symptoms over time than attending parents. There was no such effect on child depression. However, parents who attended sessions reported significantly higher depression symptoms than non-attendees at baseline. Further, attending more parent sessions did not significantly impact either symptom measure.

**Discussion:**

Given the non-significant differences of parental attendance in this study, future studies could examine less resource demanding interventions for children with emotional difficulties. When the child is struggling with anxiety and depression, the parent’s role in child’s life could be vital for symptom amelioration. The challenge is finding effective, evidence-based methods to involve parents, to reduce child emotional difficulties and improve their quality of life.

**Conclusions:**

In this preventive study, attendance in parent sessions has limited effect on parent-reported symptoms of child emotional difficulties.

## Introduction

Family, carers, and parents are crucial for children’s development in all aspects of life, also in health-related matters such as emotional difficulties [1–4]. Symptoms of anxiety and depression are associated with impairment in several arenas in life, such as family, school and social domains [5] and impacts the child`s school functioning and interpersonal relations [4]. Further, families faced with child impairment can be less motivated to attend and follow-up child treatment [6], as there are struggles dealing with everyday life related to school, friends and family [7]. At the same time, the extensive impact of parents’ support in prevention and treatment of mental health problems in children is obvious. The impact of emotional difficulties and impairment on children stresses the need for preventive interventions targeting children’s symptoms of anxiety and depression.

Limited resources in services result in a lack of treatment capacity, meaning that treatment is not always available for the target group. This underscores the need for earlier, prevention-focused efforts [8]. Ideally, prevention commences before any disorder or impairment has manifested. A recent review and meta-analysis [9] indicates that preventive interventions for youth anxiety and depression are available. However, results show small differences in symptoms between intervention and control groups post intervention. In addition, targeting at-risk children in indicated preventive interventions yielded greater effect-sizes than universal efforts, targeting all children. Also, preventive interventions may be more resource-demanding for primary care services short-term [10] as they usually involve a wider, more various target group and several professionals over a longer period compared to treatment interventions.

A systematic review of cognitive behaviour therapy (CBT) for children with depression found that parental involvement is no more effective in reducing depression than control conditions [11]. Surprisingly, some studies found that no parental involvement were significantly more beneficial for the children than control condition. Stark et al. [12] underline that although few studies find significant effects of parental psychoeducational involvement in treatment of children’s depression, parents are highly influential in their child`s core beliefs which may lead to depression.

Wei & Kendall [13] found that parental involvement in anxious children had little or no effect on symptom remission. In their article, they addressed several components that could lead to a more targeted and effective approach for parental involvement in CBT for youth anxiety in their systematic review. A study by Hudson et al. [14] indicates that for parental involvement to significantly surpass the effects of child therapy alone on anxiety, it must be highly intensive. They argue that such intensity can be achieved by including contingency management strategies (CM), such as use of attention and rewards to shape non-anxious behaviour [15] and the transfer of control (TC) which means that expert knowledge and skills are transmitted from the group leader to the parents who will transport these to the child [16]. Tentative support for this mechanism has been provided by Khanna and Kendall [17] which can promote more adaptive parental behavior for children with emotional difficulties. These findings help clarify the mixed results of effectiveness trials of parental participation in CBT interventions for anxious and depressed children [12, 18]. These results were supported in the current preventive ECHO-trial, where the effect of parent sessions (high involvement) did not surpass a psychoeducational brochure (low involvement) [19]. There were statistically significant reductions in both child and parent-reported children’s anxiety and depression scores from baseline to post-intervention for children in both experimental conditions [19]. However, there was no statistically significant difference in effects between children in the high or low parental involvement conditions. Between post-intervention and one-year follow-up, anxiety symptoms continued to decrease in both conditions [20]. However, depression scores continued to decline in the parent sessions but remained stable in the brochure condition for both child and parent-report. Attendance data from parent sessions suggests that parents attended a varying number of sessions (0–5 sessions). Based on the findings in the ECHO-trial and varying degree of participation in the high involvement condition, we aimed to explore the impact of attendance in parent groups on parent-reported child symptoms of anxiety and/or depression. We hypothesized that greater attendance is associated with lower levels of child symptoms over time.

## Methods

### Study design

This study is part of a larger trial, the ECHO-trial. With a cluster randomized factorial design, we investigated three candidate components for optimizing the indicated preventive CBT intervention, EMOTION, for anxious and sad 8–12-year-olds, illustrated in Table 1. See Neumer et al. [21] for more details on the trial design.

**Table 1:** The conditions in the ECHO-trial (in a cluster randomized factorial design)

Condition	MFS App	Delivery format	Parent Involvement
1	Yes	Blended	High
2	Yes	Blended	Low
3	Yes	Group	High
4	Yes	Group	Low
5	No	Blended	High
6	No	Blended	Low
7	No	Group	High
8	No	Group	Low

MFS = Multi Feedback System

Blended = 8 physical (group) sessions + 8 digital (individual) sessions

Group = 16 physical group sessions

High = 5 parent group sessions

Low = Psychoeducational brochure

In this study, the sample is drawn from the high parental involvement condition. In the ECHO-trial, parental involvement was tested in two levels: low parental involvement where parents received a psychoeducational brochure to guide them how to assist their child, and high parental involvement, consisting of 5 group sessions. In the high parental involvement condition, three out of the five group sessions were together with their child, following the sixteen child sessions (blended or group) over 8–10 weeks. Sessions focused on the family doing positive activities together, positive parenting, and how to support the child in exposures and behavioral experiments. The parental group sessions had the following content:

Session 1: Motivation, setting goals, facilitate parent-child relationship

Session 2: Positive parenting and reinforcement (with child)

Session 3: Cognitive-behavioral model, behavioural experiments, recognition of emotions

Session 4: Cognitive restructuring, behavioural experiments, engagement in problem solving (with child)

Session 5: Closing session, parental modeling behaviour (with child)

Parents received a workbook to apply in the groups and as homework. The group leaders were equipped with a parent session manual and recorded attendance after each session.

### Procedure and participants

Children and parents from 58 schools across Norway participated in the ECHO-trial. Children with valid consent (*n* = 1364) completed a screening for symptoms of anxiety and depression. Children with a self-reported score of ≥ 1 standard deviation above the expected mean for depressive and/or anxious symptoms [22–25] were invited to EMOTION groups (*n* = 756).

From schools in the high involvement condition, 302 children and their parents accepted participation. The present study includes 385 parents of 235 children who responded to one or more surveys at baseline, post-intervention or one-year follow-up, and whose attendance was documented by group leaders. Non-attendance was not recorded in all cases where one parent was present and not the other, resulting in 61 cases with missing data.

Procedures in this study complied with the Helsinki Declaration, and was approved by the Regional Committees for Medical and Health Research Ethics (REK) - South East Norway (2019/1198) and The Norwegian Agency for Shared Services in Education and Research (Sikt) (152745). The ECHO study was registered with clinicaltrials.gov (NCT04263558), first posted on February 11, 2020, last updated on November 29, 2023. The study protocol by Neumer et al. [21] describes the study and procedures detailed.

### Measures

**Demographic variables**. Child age and sex were collected in the consent form. More extensive information about the child, parent and family was collected from parents the first time they answered the survey.

**Multidimensional Anxiety Scale for Children Parent-report (MASC-P)** [26], is a 39-item parent-report scale to assess 8–19 year old’s anxious symptoms. Responses range from 0 (never), 1 (rarely), 2 (sometimes), 3 (often). The sum of scores gives a total anxiety score. Previous studies have reported good psychometric properties [27]. In this sample internal consistency reliability was excellent measured by McDonald’s Omega (ω) (*n* = 345, ω = 0.89) at baseline.

**The Mood and Feelings Questionnaire – Short form Parent-report (SMFQ-P)** [28], is a 13-item parent-report scale to assess 8–18-year old’s depressive symptoms. Responses range from 0 (not true), 1 (sometimes true) to 2 (true). The total score range is 0 to 26. Psychometric properties have been established in Norway, with good internal consistency for both a population sample and a sample of children with elevated symptom levels [29, 30]. In this sample, internal consistency reliability was good (*n* = 342, ω = 0.87) at baseline.

**Columbia Impairment Scale Parent Version (CIS-P)** [31] is a 13-item parent-report scale assessing children’s general impairment in various functional domains, including relations with family members at home, relations with peers, school functioning, and involvement in general interests and activities. Items are scored ranging from 0 = “no problem”, 1–3 = “some problem” to 4 = “very bad problem”. Not-applicable/do-not-know responses are scored using a 5 and treated as missing data in the analysis. Sum scores are calculated using individual mean level imputation, which accounts for missing data. A score of ≥ 15 is considered cut-off for impairment [31]. The CIS-P has shown good psychometric properties [4, 31]. In this sample, internal consistency reliability was adequate (*n* = 270, ω = 0.84) at baseline.

**Dose** was operationalized by parents’ attendance in two dose variables. This information was provided from group leaders after each session. First, parents who did not attend in any session were categorized as non-attendees, whereas parents who attended one or more sessions were categorized as attendees. Second, we included the number of attended sessions beyond one as a scalar variable.

### Statistical analyses

We used linear mixed effects models with anxiety (MASCP) and depression (SMFQP) one at a time as dependent variable. We included attendance and time as categorical covariates, and their interaction. We included random effects of families nested within schools. The child’s level of impairment has been shown to affect both parent’s help seeking behaviour and treatment effect in children, thus, in a second model, we adjusted for child impairment (CIS-P) at baseline in addition to age, sex, family income, and parent´s education. The models include parents with partly missing data, and results are unbiased if data is missing at random (MAR), while a complete case analysis is unbiased only under the more restrictive missing completely at random (MCAR) assumption. We carried out the analyses in two ways, first the independent variable dichotomized as attendance versus non-attendance, and second also including the number of attended sessions beyond one as a scalar variable. We regarded two-sided *p*-values under 0.05 to represent statistical significance. Analyses were carried out in SPSS28 and Stata17.

## Results

### Parent and child characteristics

Parent and child demographics are presented in Table 2. In this sample of parents, 58% (*n* = 225) were mothers, with a mean age of 41.7 years. Both parents were invited, and the 385 participants had 235 unique children who participated in this study. Most participants were parents of girls (58%). 68% of parents had tertiary education. Parents on average attended in 3.1 parent sessions, and only 9% of the sample did not attend at all. The child’s parent-reported functional impairment score (CIS-P) at baseline was M = 13.1 (SD = 8.0), which indicated that the child’s overall functioning on average was below the scale cut-off of impairment [31]. See Table 2.

**Table 2:** Parent/carer and child sample characteristics (*n* = 385)

Parent/carer characteristics	*N*	%
**Age in years** (mean, SD)	41.7 (5.8)	
**Relationship to child**		
Mother	225	58%
Father	153	40%
Other	7	2%
Fosterparent	(6)	(2%)
Step parent	(1)	(not counted for)
**Birthplace**		
Norwegian	353	92%
Other	32	8%
**Employment**		
Full time employed	307	80%
Part time employed	40	10%
Currently not working	38	10%
**Completed education**		
Lower secondary school	53	14%
Upper secondary school	73	19%
University/College ≤ 4 years	130	34%
University/College > 4 years	129	34%
Family gross income*		
Less than 200.000 NOK	2	1%
201.000 to 350.000 NOK	14	4%
351.000 to 500.000 NOK	26	7%
501.000 to 800.000 NOK	72	19%
801.000 to 1 million	79	21%
Over 1 million	192	50%
**How would you describe your income**		
Poor	5	1%
Average	102	27%
Good	212	55%
Very good	66	17%
**Attendance in parent sessions**		
5 sessions	82	21%
4 sessions	101	26%
3 sessions	78	20%
2 sessions	55	14%
1 session	35	9%
No sessions	34	9%
**Child gender**		
Parent of daughters	224	58%
Parent of sons	161	42%
**Child Age** (mean years, SD)	10.5 (0.7)	
**MASC-P baseline (T1) (*n* = 346)** (mean, SD)	51.6 (15.0)	
**SMFQ-P baseline (T1) (*n* = 342)** (mean, SD)	6.7 (5.0)	
**CIS-P – baseline (T1) (*n* = 342)** (mean, SD)	13.1 (8.0)	

*100 NOK = 8.8 EURO/19.4 USD/ 7.4 GBP (per June 2024)

**Fig. 1:**
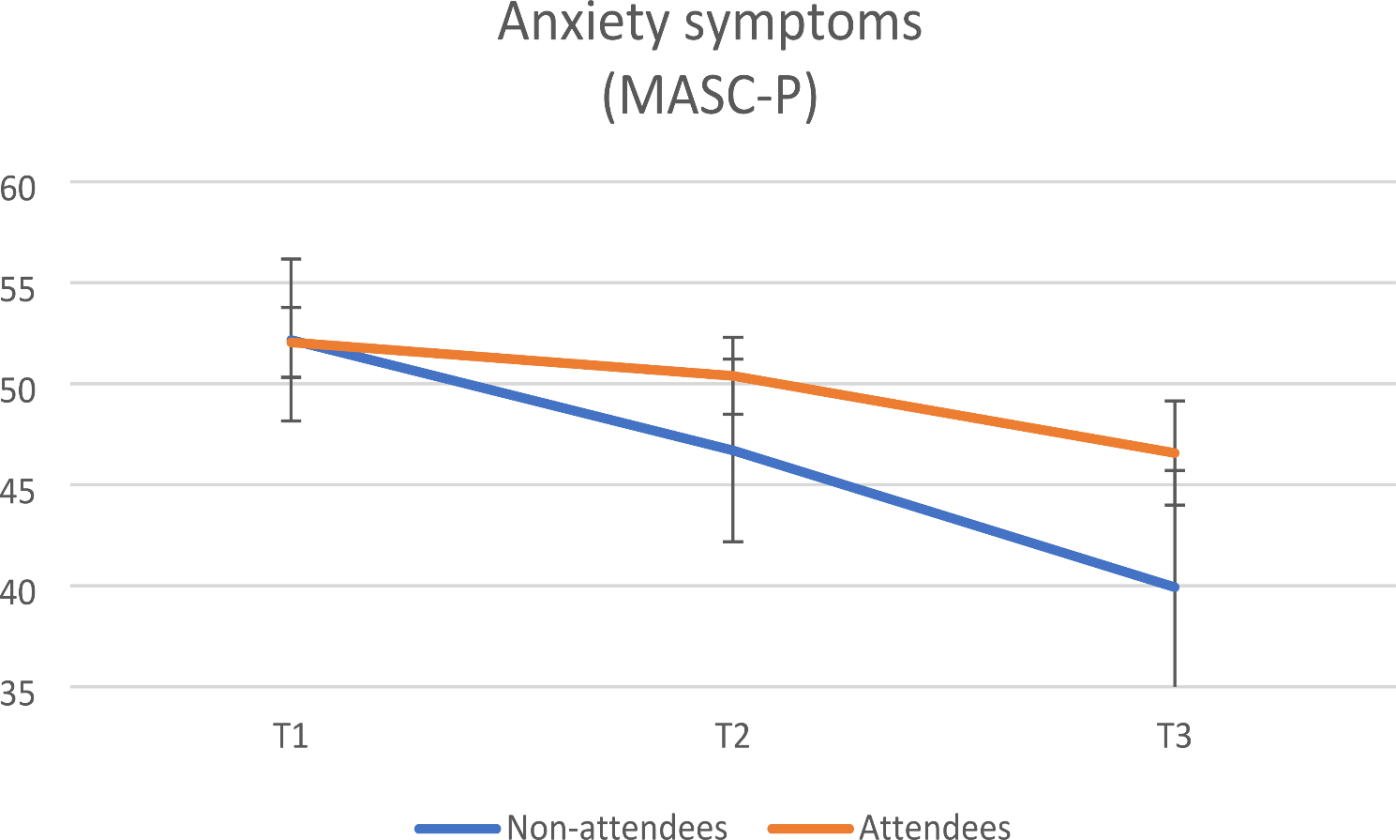
Parent-reported anxiety scores (MASC-P) for children attending and non-attending parents from the high involvement component, at all three measurement points with 95% confidence intervals (*N* = 385)

**Fig. 2:**
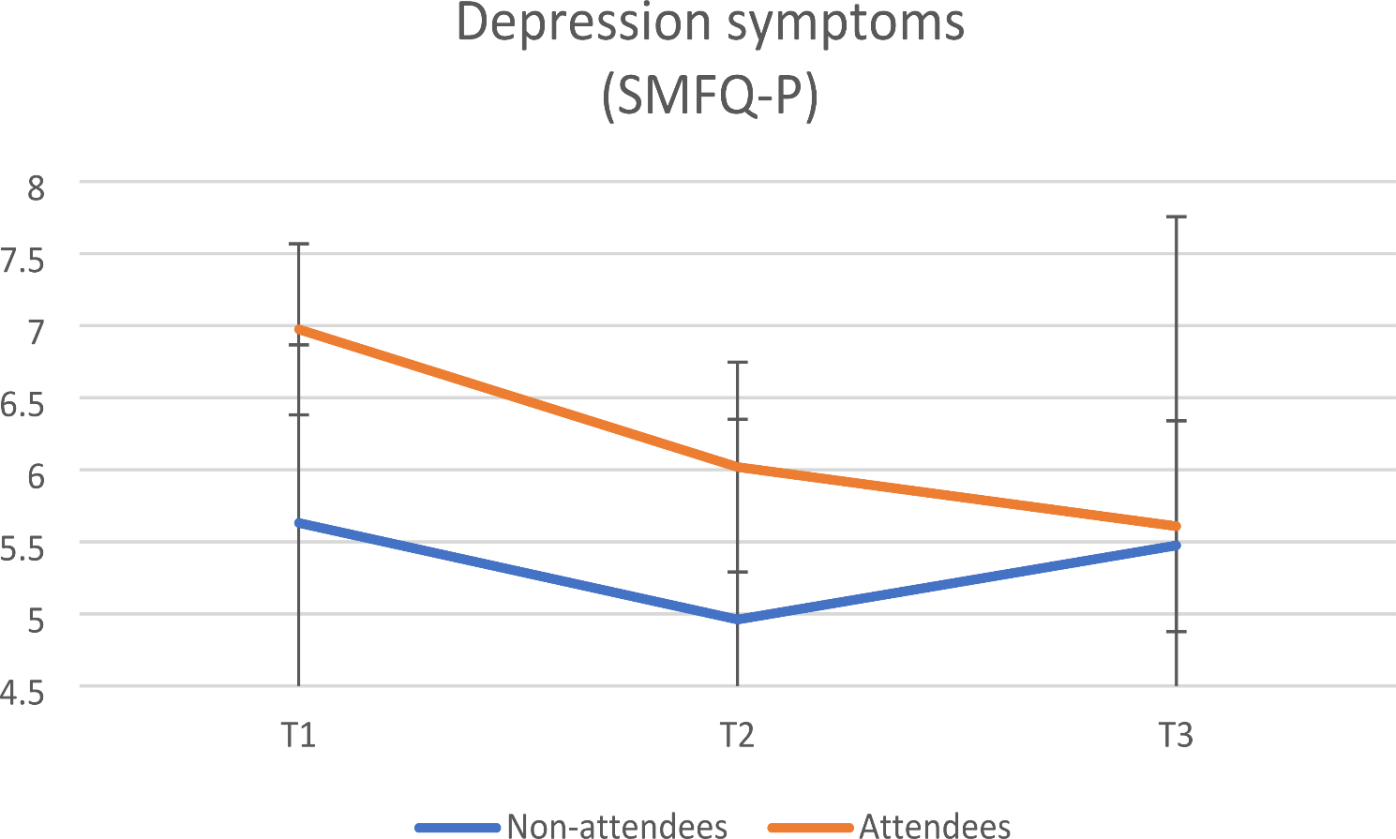
Parent-reported depression scores (SMFQ-P) for children of attending and non-attending parents from the high involvement component, at all three measurement points with 95% confidence intervals (*N* = 382)

Figures 1 and 2 illustrate the development of parent-reported child symptoms of anxiety and depression at baseline (T1), post intervention (T2) and one-year follow-up (T3). Both parent groups reported similar symptom levels for child anxiety at baseline, and a reduction from baseline to post intervention and one-year follow-up. Parents who attended sessions reported significantly higher child depression symptoms, than non-attendees at baseline (*p* = 0.030). However, there were no significant difference in parent-reported depression symptoms over time.

**Effects of attendance on parent-reported child anxiety and depression**. The results from linear mixed models of attendance vs. non-attendance over time on parent-reported symptoms of child anxiety and depression are shown in Table 3. Non-attendees reported significantly greater reduction in child anxiety symptoms over time compared to attendees. There was no statistically significant effect of parental attendance over time on child depression. However, non-attending parents reported significantly less depression symptoms than attending parents at baseline. Adjusting for child impairment at baseline, age, sex, family income and education resulted in substantially the same results. To assess whether number of attended sessions affected parent-reported symptoms over time, we added attendance beyond one session as a covariate in a second analysis, The results were substantially the same for anxiety (MASC-P) as well as for depression (SMFQ-P), data not shown.

**Table 3:** The effect of attendance vs. non-attendance (interaction with time) on parent-reported child anxiety (MASC-P) and depression (SMFQ-P) in linear mixed models that were unadjusted and adjusted for child impairment (CIS-P) at baseline, age, sex, parental income, and education

	T1-T2 (Baseline to post-intervention)			T1-T3 (Baseline to one-year follow-up)		
	Estimate	95% CI	*p*	Estimate	95% CI	*p*
**MASC – P (** ***n*** ** = 385)**						
Unadjusted	3.81	0.75 to 6.86	0.015	6.76	2.09 to 11.44	0.005
Adjusted	3.80	0.80 to 6.81	0.013	5.47	0.61 to 10.33	0.027
**SMFQ-P (** ***n*** ** = 382)**						
Unadjusted	-0.29	-2.00 to 1.43	0.75	-1.21	-3.68 to 1.26	0.34
Adjusted	-0.44	-2.21 to 1.32	0.62	-2.15	-4.80 to 0.49	0.11

## Discussion

In this study on the impact of attendance in parent group sessions in an indicated, preventive intervention, non-attendees reported a significantly greater reduction in child anxiety symptoms over time compared to attendees. There was no statistically significant effect of attendance over time on symptoms of child depression. There was no additional effect of attendance beyond one session, neither for anxiety nor depression.

The lack of positive effects of attendance on child amelioration is surprising. We expected that the competencies provided in the parent sessions would enhance parental practices such as communicating about feelings, coping strategies and problem solving strategies [30]. We also assumed that participating in sessions would increase parental assistance in homework assignments related to child group sessions. Therefore, we expected larger symptom reductions in attendees [32]. Parent sessions may have given the parents a new understanding of their child’s feelings and reactions, making them more attentive to typical anxious behaviors, and increased parental awareness [13]. In turn, this could have led to higher parent-reported child anxiety. If this is the case, the parent sessions might not have enhanced important skills to deal with these struggles, but left parents able to identify, but not help their child [33]. This would be in line with Hudson et al. [14] recommendations about increasing intensity, through contingency management (CM) and/ or transfer of control (TC), to promote parental behaviour related to the child`s anxiety.

Interestingly, attending parents reported significantly higher child depressive symptoms at baseline, than non-attending parents. One plausible explanation could be that parents who report higher child depression symptoms are more motivated to participate in this intervention. When your child struggles you might be more motivated to seek out interventions that increase your ability to help.

For depressive symptoms, there were no significant differences over time between parents who did or did not attend. A possible explanation could be that depressive symptoms and expressions are more difficult for parents to recognize. For instance, a sad child may be restless and angry. This may be misinterpreted by parents, which may have led them to react maladaptively [34]. Also, reports from non-attending parents on their child’s depressive symptoms showed a reduction post-intervention (T2) but indicated an increase at follow-up one year later (T3) compared to baseline levels. This can be interpreted as non-attending parents not being equipped with the skills to help their child after the intervention ended, leading to a return of depressive symptoms to pre-intervention levels. For children with depressive symptoms, relational protective factors such as self-efficacy, family climate, and social support can influence the development of depressive symptoms over time [35]. In contrast, attending parents continued to report lower levels of their child’s symptoms of depression at the one-year follow-up. This suggests that the relational protective factors may have improved through the competencies gained from attending parent sessions.

Parents opportunities to support their child is strongly associated with their socioeconomic status [36]. Higher socioeconomic status can contribute to various types of capital [37], enabling parents to increase and improve their ability to spend time and be involved in their child’s life. With regards to education and income, our samples self-reported socio-economic status was high, compared to the Norwegian population [38, 39].

Parents also reported relatively low child impairment at baseline. Together with the elevated SES, this supports how social and financial capital [37] can contribute to adaptive parenting [36] and thereby improved health [19]. Further, adjusting for the child`s impairment, age, gender, and parental socio-economic status did not alter the results substantially.

Families in this study participated in an indicated preventive intervention, and the sample was selected based on child’s elevated self-reported MASC and SMFQ scores. Thus, parent-reported symptoms are, as expected, higher than comparable community samples, with mean SMFQ-*P* = 3.1 [24] and MASC-*P* = 33.5 [40], considerably lower than baseline scores in this study, which were 6.7 and 51.6, respectively.

Given these results, future studies should examine whether less resource demanding interventions, for example digital self-help interventions for parents is an effective alternative [41] although some studies on parental involvement reveal less promising effects [42]. Furthermore, if we are to retain parental involvement in prevention of anxiety and depression, research to add to the effect of child alone interventions are necessary, given the scarce resources in services.

Involving parents when working with children’s emotional problems should enhance the effects on symptoms long-term, producing less suffering and relapse through improved understanding of parents and altered parental practices [12, 43]. In the long run, lasting improvement and better lives would be the outcome for the children, and reduced community costs. To achieve this, parent sessions should be better tailored to the family and the specific child’s problems, thus, increasing intensity of the intervention [18, 44]. One alternative approach could be to target the proposed mechanisms of change in parent components [13, 14].

Including a separate parental involvement component to child-focused interventions often requires substantial additional time and resources from both healthcare services and families. Considering the high demand from the public and politicians to secure effective and cost-effective health care services, and an increasing expectation that families contribute to their own well-being, the parent component of the EMOTION intervention should be critically considered.

### Strength and weaknesses

The high number of participants and sophisticated design in the ECHO-factorial trial, including urban and rural schools with group leaders from primary health services and pedagogical staff, the inclusion and selection process, and the low drop-out rates are all strengths of the study. The current sample (*n* = 385), however, consist only of parents who answered our survey and had at least one registration from group leaders of being absent or present in parent sessions. The study is executed in a non-clinical context and participants might have had limited motivation for answering questionnaires. Not all group leaders registered absent parents if the other parent attended, which means that we excluded some parents who had answered the questionnaire, but we were uncertain if they had attended sessions. Importantly, our sample is skewed towards well educated, employed parents, where 72% report their financial situations as good/ very good. Higher SES is considered a protective factor against psychopathology symptoms in children [45]. This limits the generalizability of our results. The current results are based solely on parent-reported child symptoms. Parents may have difficulties estimating internalizing symptoms in their child, or their reports may be biased due to their own mental health issues [46] and should be considered.

Lastly, the study was conducted during the Covid-19 pandemic, and it is uncertain how this extraordinary situation may have influenced the results. However, the results from the first wave, which was executed during the initial outbreak and lock-down, March 2020 – May 2020, was excluded and considered a pilot trial as groups and parent sessions were not executed as described in the manual, i.e. on teams, outdoors or divided in smaller cohorts.

## Conclusions

In this indicated preventive study, attendance in parent sessions has limited effect on parent-reported symptoms of child emotional difficulties over time. Dose does not matter.

Authors’ contributions.

All authors have read and provided substantial contributions to the final version of the manuscript. EVB and KY has drafted and developed the study and been responsible in all parts of the process. KY is Ph.D. candidate and responsible for statistical procedures and analysis of data, overseen by SL. SPN is the PI of the ECHO-trial and JMI is the local PI for the project and has contributed to all phases of the study.

## Data Availability

Data is not publicly available due to privacy restrictions but can be made available upon reasonable request to senior authors. Data that support the findings of this study have been stored at Services for sensitive data (TSD), University of Oslo, Norway.
